# Effects of Noninvasive Low-Intensity Focus Ultrasound Neuromodulation on Spinal Cord Neurocircuits In Vivo

**DOI:** 10.1155/2021/8534466

**Published:** 2021-11-27

**Authors:** Ye-Hui Liao, Mo-Xian Chen, Shao-Chun Chen, Kai-Xuan Luo, Bin Wang, Yao Liu, Li-Juan Ao

**Affiliations:** ^1^School of Rehabilitation, Kunming Medical University, Kunming 650500, Yunnan Province, China; ^2^Department of Orthopaedics, Affiliated Hospital of Southwest Medical University, Luzhou 646000, Sichuan Province, China

## Abstract

Although neurocircuits can be activated by focused ultrasound stimulation, it is unclear whether this is also true for spinal cord neurocircuits. In this study, we used low-intensity focused ultrasound (LIFU) to stimulate lumbar 4–lumbar 5 (L4–L5) segments of the spinal cord of normal Sprague Dawley rats with a clapper. The activation of the spinal cord neurocircuits enhanced soleus muscle contraction as measured by electromyography (EMG). Neuronal activation and injury were assessed by EMG, western blotting (WB), immunofluorescence, hematoxylin and eosin (H&E) staining, Nissl staining, enzyme-linked immunosorbent assay (ELISA), immunohistochemistry (IHC), somatosensory evoked potentials (SEPs), motor evoked potentials (MEPs), and the Basso–Beattie–Bresnahan locomotor rating scale. When the LIFU intensity was more than 0.5 MPa, LIFU stimulation induced soleus muscle contraction and increased the EMG amplitudes (*P* < 0.05) and the number of c-fos- and GAD65-positive cells (*P* < 0.05). When the LIFU intensity was 3.0 MPa, the LIFU stimulation led to spinal cord damage and decreased SEP amplitudes for electrophysiological assessment (*P* < 0.05); this resulted in coagulation necrosis, structural destruction, neuronal loss in the dorsal horn by H&E and Nissl staining, and increased expression of GFAP, IL-1*β*, TNF-*α*, and caspase-3 by IHC, ELISA, and WB (*P* < 0.05). These results show that LIFU can activate spinal cord neurocircuits and that LIFU stimulation with an irradiation intensity ≤1.5 MPa is a safe neurostimulation method for the spinal cord.

## 1. Introduction

Neurostimulation technology, including focused ultrasound stimulation, transcranial magnetic stimulation (TMS), deep brain stimulation (DBS), and optogenetic stimulation, has become an important neuromodulation method for various neurological conditions. TMS is a noninvasive or minimally invasive neurostimulation technique, which has been widely used for neuromodulation. TMS has advantages for superficial brain regions, while its effects on deeper brain regions are limited given its poor spatial resolution [1]. DBS, including electrical stimulation or epidural electrical stimulation, is a commonly used neurostimulation method. Epidural stimulation of the spinal cord plays a positive role in the functional recovery of the injured spinal cord [2, 3]. However, because of the highly diffuse electric field and lower spatial resolution, it is difficult to locate a specific area of interest using electrical stimulation [4]. Moreover, surgery is also required, and the electrode is inserted in or on the cerebral cortex or epidural space for electrical stimulation [2]. Optogenetic stimulation also requires invasive procedures and genetic manipulation, which are not feasible in humans [5, 6]. Recently, focused ultrasound has attracted much attention and interest due to its high spatial resolution, noninvasive neurostimulation, and effective stimulation of the deep tissues with submillimeter static resolution [7, 8]. Consequently, it has become an alternative modality for neuromodulation [9].

Ultrasound is a mechanical pressure wave with a frequency of >20 kHz, which can be transmitted through bone and soft tissues. An acoustic intensity <500 mw/cm^2^ (low-intensity ultrasound) has shown significant biological effects without producing thermal effects or tissue damage [10, 11]. Moreover, many studies have confirmed that low-intensity ultrasound stimulation can inhibit or stimulate neurons both in vitro and in vivo. Recent research has shown that low-intensity, low-frequency ultrasound stimulation of hippocampal slices excites the neurons and network activity by activating voltage-gated sodium and calcium channels [12, 13]. Further studies have confirmed that low-intensity focused ultrasound (LIFU) activates neurocircuits in both model organisms and humans. Indeed, pulsed focused ultrasonic stimulation has been shown to effectively induce nerve responses and action potentials in the giant fibers of invertebrate animals [14, 15]. Moreover, precise stimulation of the deep brain nuclei and modulation of brain neuronal activity [16–19] have been shown to induce muscle contraction of the limbs of rats and rabbits, as verified by electromyography (EMG) [20, 21]. Primate studies have also found that focused ultrasound stimulation of the brains of two awake macaque rhesus monkeys significantly modulates high-level cognitive behavior [22]. Gavrilov [23] confirmed that ultrasound could induce tactile, thermal, and pain sensations by activation of somatosensory neurons in humans. As a result, low-intensity ultrasound, especially LIFU, has gained widespread attention as a potential clinical neuromodulation technology.

The spinal cord contains complex neurocircuits, the stimulation of which is an important method to treat chronic spine-related conditions, such as failed back surgery syndrome, complex regional pain syndrome, painful diabetic neuropathy, and spinal cord injury [3, 24–28]. Recently, there has been growing interest in ultrasound neuromodulation; however, little is known about whether spinal cord stimulation with LIFU can activate or inhibit spinal cord neurocircuits and whether such stimulation causes injury to the spinal cord. Activation of the spinal cord neurocircuits can induce muscle contractions and produce action potentials, which can be measured by EMG [20]. In this study, LIFU was used to stimulate the L4–L5 segments of the spinal cord in Sprague Dawley (SD) rats, and EMG was used to measure the stimulation success of the spinal cord neurocircuits. Hematoxylin and eosin (H&E) staining, Nissl staining, and biomarker tests were used to evaluate the safety of LIFU stimulation.

## 2. Materials and Methods

### 2.1. Animals

Adult male SD rats weighing 220–300 g were acquired from the Kunming Laboratory Animal Center for experimental use. All of the animal protocols were approved by the Animal Ethics Committee of Kunming Medical University (KMMU2020352). All rats were housed at a temperature of 25 ± 2°C with a 12/12-h light/dark cycle, and all rats had free access to food and water.

### 2.2. Experimental Protocol

**Figure 1:**
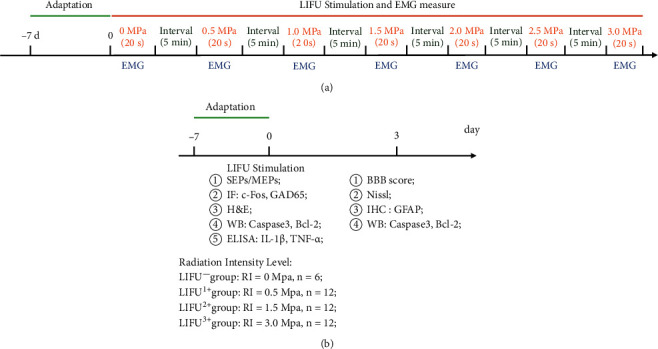
Timeline of study I (a) and II (b) experimental protocols. The rats were killed, and the safety was examined on days 0 and 3 post-low-intensity focused ultrasound (LIFU) stimulation.

After 1 week of adaptation, the SD rats were used for the experiment. The study comprised two protocols. The first study (Study I, *n* = 6) was designed to test the activation of the spinal cord neurocircuits by LIFU stimulation. In this study, the rats were anesthetized with isoflurane (1.5%), and EMG was used to measure the recruitment of the soleus (Sol) muscle when the rats received different acoustic pressure stimulations (radiation intensity (RI) = 0 MPa (*I*_spta_ = 0 mw/cm^2^), 0.5 MPa (*I*_spta_ = 60 mw/cm^2^), 1.0 MPa (*I*_spta_ = 180 mW/cm^2^), 1.5 MPa (*I*_spta_ = 320 mw/cm^2^), 2.0 MPa (*I*_spta_ = 400 mw/cm^2^), 2.5 MPa (*I*_spta_ = 500 mw/cm^2^), or 3.0 MPa (*I*_spta_ = 600 mw/cm^2^)) with 20% duty cycle (DC). There was a 5 min interval between different ultrasonic parameter tests. The study protocol is shown in Figure 1(a).

In the second study (Study II, *n* = 42), the safety of LIFU stimulation was detected by electrophysiology, neuromotor function, H&E staining, Nissl staining, enzyme-linked immunosorbent assay (ELISA), and biomarker tests. The rats were divided into four groups as follows: the negative LIFU stimulation group (LIFU^−^ group, RI = 0 MPa, *n* = 6), the LIFU stimulation 1 group (LIFU^+1^ group, RI = 0.5 MPa, *n* = 12), the LIFU stimulation 2 group (LIFU^+2^ group, RI = 1.5 MPa, *n* = 12), and the LIFU stimulation 3 group (LIFU^+3^ group, RI = 3.0 MPa, *n* = 12). After anesthesia with isoflurane (1.5%), the rats were stimulated with LIFU for 20 min. After LIFU treatment, the safety test was performed according to the protocol shown in Figure 1(b).

### 2.3. LIFU Stimulation

**Figure 2:**
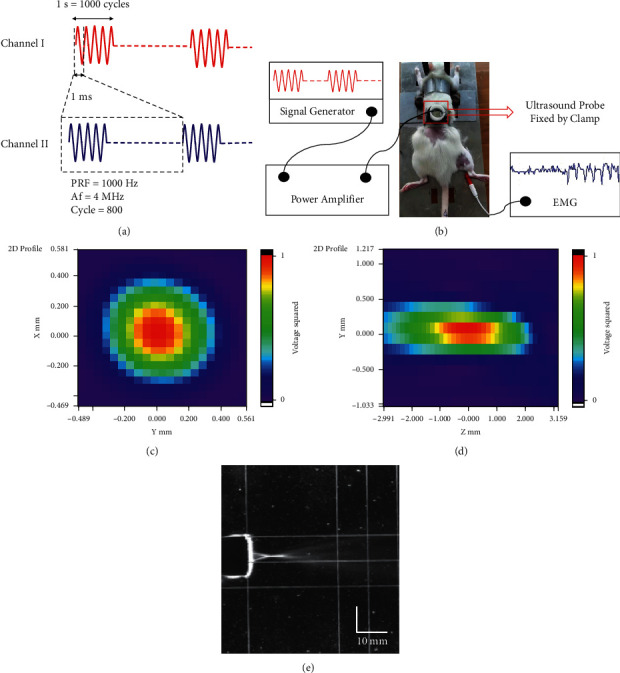
(a) Schematic of the LIFU pulsing strategy. (b) Schematic of LIFU stimulation of the spinal cord and electromyography (EMG) examination. The signal was generated by the generator, amplified by the amplifier, and then converted into an acoustic signal by the ultrasound probe. The ultrasound probe was fixed by the clamp on the back of the rats at the segment L4–L5 spinal cord level. The recruitment of the soleus muscle was recorded at the time of LIFU spinal cord stimulation. (c–e) Parameters of focal ultrasound, including the acoustic-intensity distribution map from transverse and sagittal planes.

The LIFU stimulation procedure was performed as follows [29]: after anesthesia, the rats were fixed on a table, and the hair on their backs was removed using a depilating cream to expose the L4–L5 segments of the spinal cord. An ultrasound probe was fixed to the back of the L4–L5 spinal cord segment with a clapper. An ultrasound gel was used to fill the space between the skin and the ultrasound probe. A waveform signal was generated by a two-channel function/arbitrary waveform generator (DG4202, RIGOL, China) and was amplified with a 50 W power amplifier (Dahan Radio Studio, China). The amplified signal activated the ultrasonic probe. Channel I was set to deliver the wave signal with cycles of 1000 Hz/s, a burst duration of 1 s, and a 20% total duty cycle. Channel II was set to deliver the ultrasound probe with a frequency of 4 MHz and 800 cycles for every pulse period (Figures 2(a)–2(b)). The acoustic pressure of the ultrasound was measured using a hydrophone (Onda HNP-1000, ONDA Corporation, Sunnyvale, CA, USA) in a water tank (Figures 2(c)–2(e)).

### 2.4. Neuromotor Function Assessment

The Basso–Beattie–Bresnahan locomotor rating scale (BBB scale) was used for the hind limb neuromotor function assessment pre- and post-LIFU. The assessment was performed as previously described [30]. The BBB rating scale assesses the coordination of limb movement, paw placement, and tail balance. The scale ranges from 0 to 21 points, where 0 is defined as no visible movement of the legs, and 21 is defined as normal neuromotor function, i.e., the rat can walk continually on the paws, with consistent plantar stepping, coordinated gait, trunk stability, a cocked tail, and parallel throughout the stance.

### 2.5. Electrophysiology Test

The recruitment of Sol muscle was used to assess the activation of spinal neurocircuits and was measured by EMG using an electromyographic evoked potentiometer (Neuropack® S1 MEB-9400, Nihon Kohden, Japan). EMG was conducted as described previously [31, 32]. Briefly, the EMG signal was measured by a concentric circular electrode that was percutaneously inserted into the Sol, and the reference electrode was percutaneously inserted into the tail. The EMG signal was recorded at the same time as the LIFU stimulation of the spinal cord and was filtered at 200 Hz–5 kHz (Figure 2(b)). The amplitude (intensity of recruitment of Sol) was measured between consecutive peaks, i.e., from the positive peak to the neighboring negative peak (*μ*V).

Somatosensory evoked potentials (SEPs) and motor evoked potentials (MEPs) were used to assess the conduction function after the white and gray matter injury of the spinal cord [33]. For the SEP test [34, 35], the recording electrode was placed on the left sensorimotor cortex, the reference electrode was inserted under the skin of the nose, the stimulation electrodes were inserted under the skin of the right ankle, and the ground needle electrode was percutaneously placed in the tail. The stimulation involved sine impulses with a trigger frequency of 2 Hz and an intensity of 3 mA. Signal data (including the latency and amplitude) were recorded from the electrode placed on the sensorimotor cortex and were filtered with a bandpass (10–2000 Hz); we recorded 100 evoked potentials on average for two times with 5 min interval. The latency was defined as the time (ms) between the onset of the stimulus artifact and the first peak (positive or negative). The amplitude (*μ*V) was measured from the positive peak to the negative peak.

For the MEP test, the stimulation electrode was placed on the left motor cortex, the recording electrode was percutaneously inserted into the right fifth palmar interosseous muscle, and the reference and ground needle electrodes were placed as outlined for the SEP test. The stimulation involved sine impulses with an intensity of 4 mA. An analog amplifier (Model 1700 Differential AC Amplifier, AM Systems, USA) was used for amplification (100×), filtering (10 Hz–10 kHz, bandpass), and recording of MEP signals. The latency was defined as the time (ms) between the onset of the stimulus artifact and the first peak (positive or negative).

The EMG, SEPs, and MEPs data were visualized and recorded on a computer for further analysis using a software interface (Signal, Cambridge Electronics Design Ltd., United Kingdom).

### 2.6. Tissue Preparation

After neuromotor function and electrophysiology assessment, the rats were killed by an overdose of 1% sodium pentobarbital (40 mg/kg). Then, the tissues were collected for western blot, ELISA, and H&E staining, Nissl staining, immunofluorescence staining, and IHC staining. For western blot and ELISA, L4–L5 spinal cord segments were immediately collected and stored at −80°C until use. For the H&E staining, Nissl staining, immunofluorescence staining, and IHC staining, the rats were perfused with 200 mL of 0.1 M phosphate-buffered saline (PBS) and then with 200 mL PBS with 4% paraformaldehyde (pH 7.4). The L4–L5 spinal cord segments were collected, fixed in 4% paraformaldehyde overnight, dehydrated, and embedded in paraffin. Finally, transverse section slices (5 *μ*m thick) of the spinal cord were prepared for H&E staining, Nissl staining, immunofluorescence staining, and IHC staining.

### 2.7. Western Blotting

The spinal cord tissue (0.1 g) was dissected, ultrasonically homogenized, and lysed with PIPA buffer (RIPA : PMSF = 1 mL : 10 *μ*L) on ice for 30 min. After centrifugation at 12000 r/min for 20 min at 4°C, the supernatant was collected. The concentration of the total protein was quantified using a bicinchoninic acid assay (Enhanced BCA Protein Assay Kit, Beyotime, China), and all samples were equalized to 30 *μ*g/10 *μ*L. The samples (30 *μ*g total protein) were resolved by 10% sodium dodecyl sulfate-polyacrylamide gel electrophoresis (SDS-PAGE) and transferred to polyvinylidene difluoride membranes (PVDFs, Millipore, MA, USA). The membranes were blocked with 5% fat-free milk at room temperature for 2 h and incubated with primary antibodies at 4°C with gentle shaking overnight. The primary antibodies included polyclonal antibodies against caspase-3 (1 : 2000, Proteintech, USA), polyclonal antibodies against Bcl-2 (1 : 2000, Proteintech, USA), and *β*-actin (1 : 2000, Santa, USA). Following incubation, the membranes were incubated with the secondary antibody, peroxidase-conjugated AffiniPure goat anti-mouse/rabbit IgG (H + L) (1 : 2000, ZSGB-BIO, China) for 2 h at room temperature. Finally, the protein bands were visualized and quantified using enhanced chemiluminescence (Tanon, Shanghai, China) and the image processing system ImageJ (Rawak Software, Stuttgart, Germany). The protein concentrations were normalized to *β*-actin.

### 2.8. ELISA for IL-1*β* and TNF-*α*

The expression levels of the inflammatory factors IL-1*β* and TNF-*α* in the spinal cord were examined after ultrasound stimulation. Spinal cord tissue (0.1 g) was cut into pieces with ophthalmic scissors after adding precooled 0.1 M PBS (1 mL). Then, the cut tissues were placed in a glass homogenizer and homogenized on ice for 8 min. After centrifugation at 4°C and 5000 r/min for 5 min, the supernatant was obtained, and the total protein concentration was quantified by the bicinchoninic acid assay (Enhanced BCA Protein Assay Kit, Beyotime, China). The ELISA was performed in accordance with the instructions for IL-1*β* (Bioswamp, RA20020, China: http://www.bio-swamp.com/upload/file/201711/1510793514933161.pdf) and TNF-*α* (Bioswamp, RA20035, China: http://www.bio-swamp.com/upload/file/201910/1571017094579621.pdf).

### 2.9. H&E Staining and Nissl Staining

H&E staining and Nissl staining were used to assess the safety of LIFU for use in the spinal cord. The H&E staining was performed as follows: the slices were dewaxed, dehydrated, stained with H&E solution, cleared with xylene, and mounted with resin. Toluidine blue was used to stain for Nissl using the following procedure: the slices were dewaxed, rinsed with tap water, treated with toluidine blue, dehydrated, and sealed with neutral gum. Images of H&E staining and Nissl staining were captured using an optical microscope (Olympus Corporation, Tokyo, Japan). The Nissl substance was identified as navy or dark blue with a light blue or light background.

### 2.10. Immunofluorescence Staining and IHC Staining

For immunofluorescence staining and IHC staining, paraffin sections (5 *μ*m thick) were prepared. First, the sections were subjected to dewaxing, antigen repair, and H_2_O_2_ elimination of endogenous peroxidase. For immunofluorescence staining, the slices were incubated with 5% goat serum and 0.03% Triton X-100 in 0.1 M PBS for 2 h. Then, the slices were incubated with primary antibodies, including monoclonal antibodies against c-fos (1 : 200, Proteintech, USA) and GAD65 (1 : 200, CST, USA), at 4°C overnight. Following incubation, the slices were incubated with secondary antibodies, including anti-rabbit IgG (H + L), F(ab′)2 fragment (Alexa Fluor® 594 Conjugate) and anti-mouse IgG (H + L), F(ab′)2 fragment (Alexa Fluor® 488 Conjugate) at room temperature in the dark for 2 h. After 3 × 10 min washing with PBS, the sections were incubated with 4′, 6-diamidino-2-phenylindole (DAPI; Sigma, USA). The images were captured via a fluorescence microscope (Olympus Corporation, Tokyo, Japan), and ImageJ software (NIH, Bethesda, MD, USA) was used to quantify the number of positive cells.

For IHC, the slides were incubated with the primary antibody GFAP (1 : 1000, Cell Signaling, USA) at 4°C overnight. Then, the slides were incubated with secondary antibody labeled with poly-HRP anti-rabbit IgG (1 : 1; Beijing Zhongshan Golden Bridge Biotechnology) for 50 min. After staining with DAB for 20 s and counterstaining with hematoxylin for 8 min at room temperature, the images were captured via a light microscope (Olympus Corporation, Tokyo, Japan), and ImageJ software (Rawak Software) was used to quantify the density of positive regions.

### 2.11. Statistical Analyses

The data are presented as the mean ± standard error of mean (SEM). SPSS 23.0 (IBM Corp., Armonk, NY, USA) was used for all statistical analyses, and GraphPad Prism software version 8.0 (GraphPad Software Inc., San Diego, CA, USA) was used to prepare the graphs. The amplitude of EMG, the gray intensity of western blot, the number of positive cells in immunofluorescence, and the density data for IHC were calculated. After verifying that all data satisfied the normality of distribution, differences among different intensities of stimulation were determined using analysis of variance (ANOVA). When an ANOVA showed a significant difference, Fisher's protected least significant difference (LSD) tests were used for pairwise comparisons. Differences between pre- and post-LIFU stimulation were analyzed using paired *t*-tests. Two-tailed *P* values <0.05 were considered statistically significant.

## 3. Results

### 3.1. LIFU Stimulation Enhances the Recruitment of Sol Muscle

**Figure 3:**
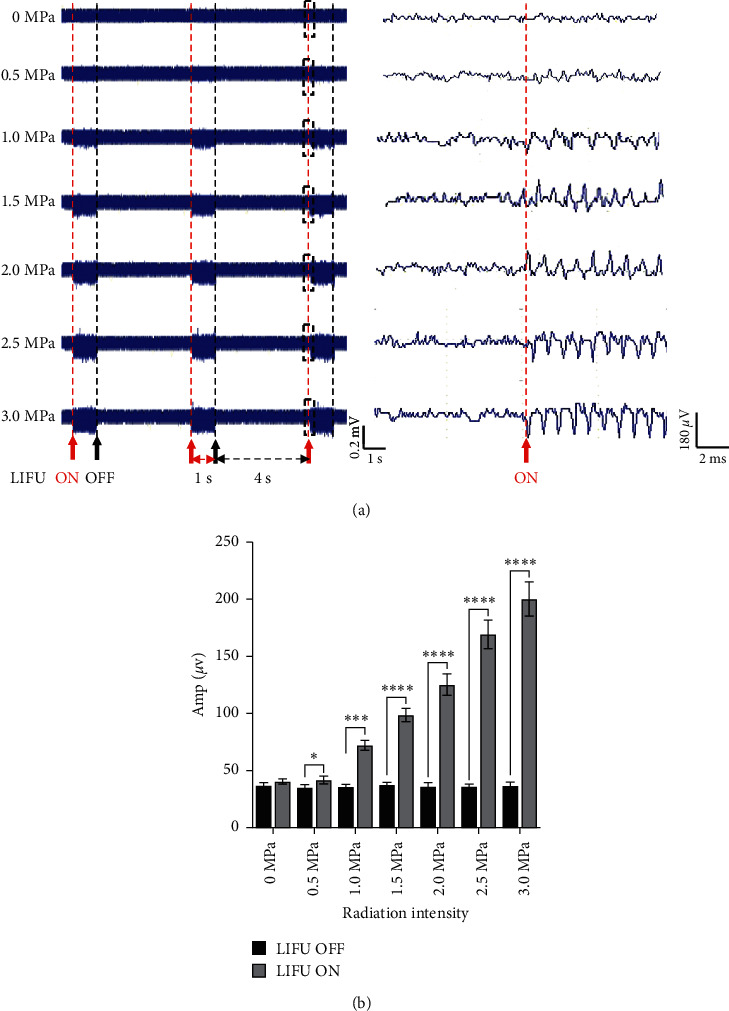
Electromyography (EMG) shows the recruitment of the soleus (Sol) muscle by different intensities of LIFU stimulation. (a) The red arrow shows the moment at which the LIFU was initiated, and the black arrow shows the moment at which the LIFU was stopped. The duration (LIFU on) from the red arrow to the black arrow was 1 s and that from the black to the red arrow (LIFU turned off) was 4 s. The black dotted rectangle shows the recruitment of Sol muscle and activation of EMG. (b) Amplitude of EMG after different irradiation intensity stimulations. ^*∗*^*P* < 0.05, ^∗∗∗^*P* < 0.01, and ^∗∗∗∗^*P* < 0.0001. Each symbol represents the mean ± SEM; paired *t-test*; *n* = 6 rats per assay.

As shown in Figure 3, LIFU simulation of the spinal cord enhanced the recruitment of Sol muscle. When the LIFU intensity was >0.5 MPa, the recruitment of Sol muscle was measured on EMG. With the increase in the stimulation intensity, the recruitment intensity (Amp, *μ*V) of the Sol muscle also increased (Supplementary Figure 1). When the stimulation intensity was >1.0 MPa, LIFU ON induced significant muscle recruitment and the EMG amplitude was significantly higher than that at LIFU OFF stimulation (*P* < 0.05). There was no significant difference in the amplitude among LIFU OFF time point (*P* > 0.05) (Supplementary Figure 1).

### 3.2. LIFU Stimulation Enhances the Number of c-fos- and GAD65-Positive Cells

**Figure 4:**
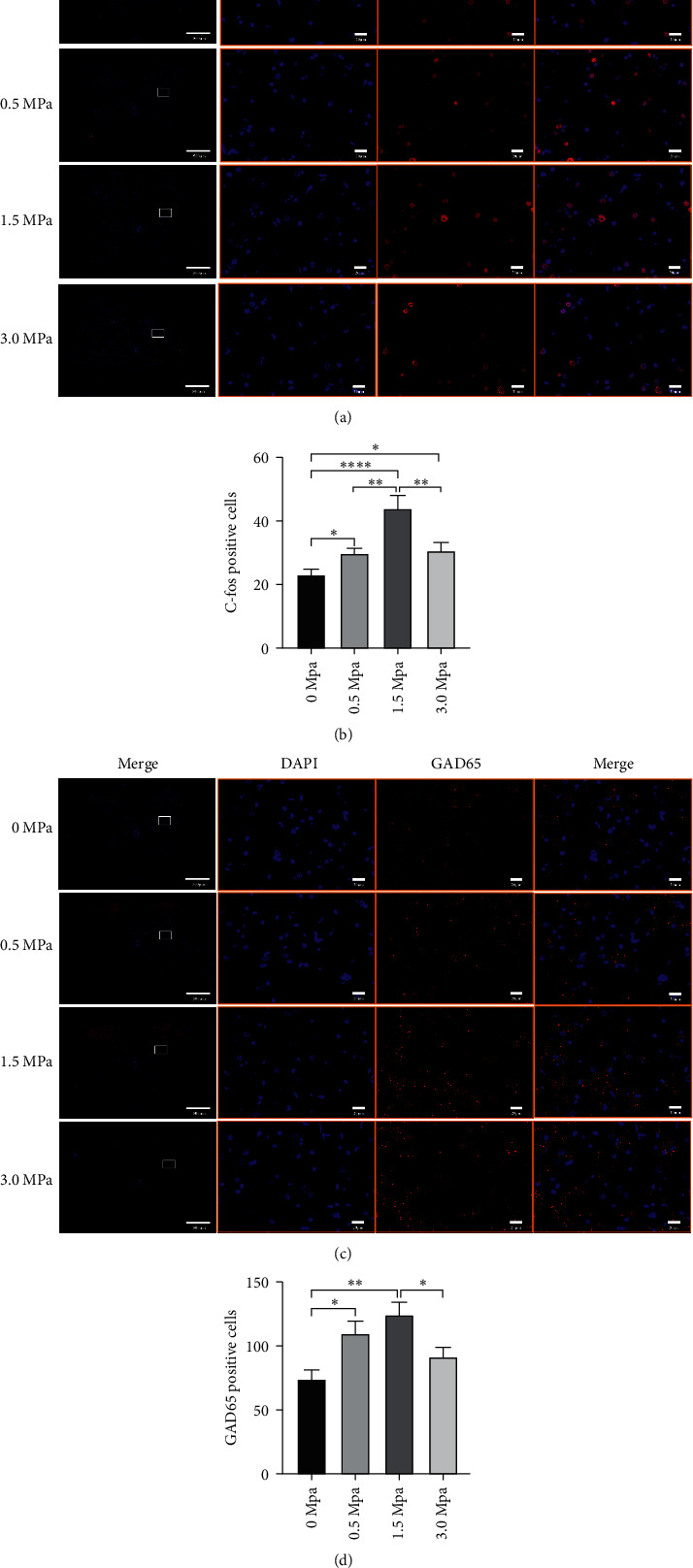
The effect of LIFU stimulation on neuron and synaptic activation in the lumbar spinal cord (×40 and ×400). Scale bar = 500 *μ*m and 20 *μ*m. (a, c) Representative immunofluorescence pictures showing the c-fos-positive (a) and GAD65-positive (c) cells after different intensities of LIFU stimulation of the lumbar spinal cord. (b, d) C-fos- and GAD65-positive cells after different intensities of stimulation. ^*∗*^*P* < 0.05, ^∗∗∗^*P* < 0.01, and ^∗∗∗∗^*P* < 0.0001. Each symbol represents the mean ± SEM; one-way ANOVA, followed by LSD test for pairwise comparisons; *n* = 3 rats per assay.

c-fos and GAD65 were used as markers of neuronal and synaptic activity, respectively. Neuronal activation can increase the number of c-fos-positive cells and GAD65-positive cells [36, 37]. We found that the numbers of c-fos-positive cells increased after 0.5 MPa, 1.5 MPa, and 3.0 MPa stimulation and that those of GAD65-positive cells increased after 0.5 MPa and 1.5 MPa stimulation compared to stimulation with 0 MPa (negative stimulation) (*P* < 0.05). However, the highest number of c-fos- and GAD65-positive cells was found in the 1.5 MPa stimulation group (Figure 4).

### 3.3. Neuromotor Function and Electrophysiological Assessment

**Figure 5:**
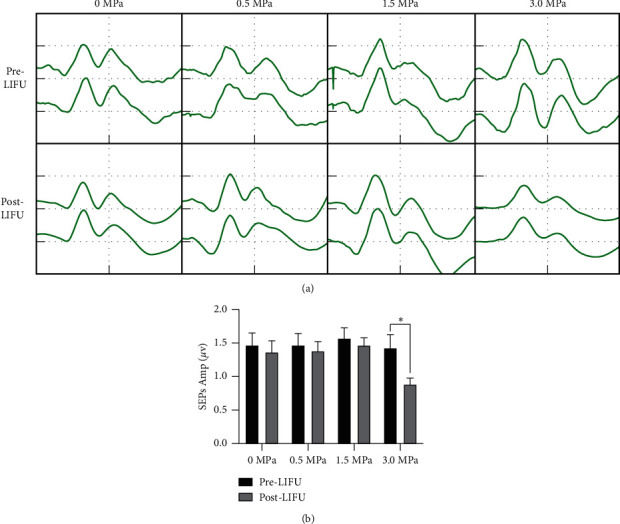
(a) Somatosensory evoked potentials (SEPs) were used to detect somatosensory conduction from the spinal cord. (b) SEP amplitude analyses for different intensities of stimulation. Each symbol represents the mean ± SEM; paired *t-test*; *n* = 6 rats per assay.

The safety of LIFU stimulation was examined at different irradiation intensities (0 MPa, 0.5 MPa, 1.5 MPa, and 3.0 MPa). After ultrasonic stimulation with different intensities, the rats showed no changes in BBB score and latency of SEPs and MEPs (Supplementary Figure 2), except that the 3.0 MPa group had decreased SEP amplitudes (Figure 5).

### 3.4. Inflammatory Factors and Histological Examination

**Figure 6:**
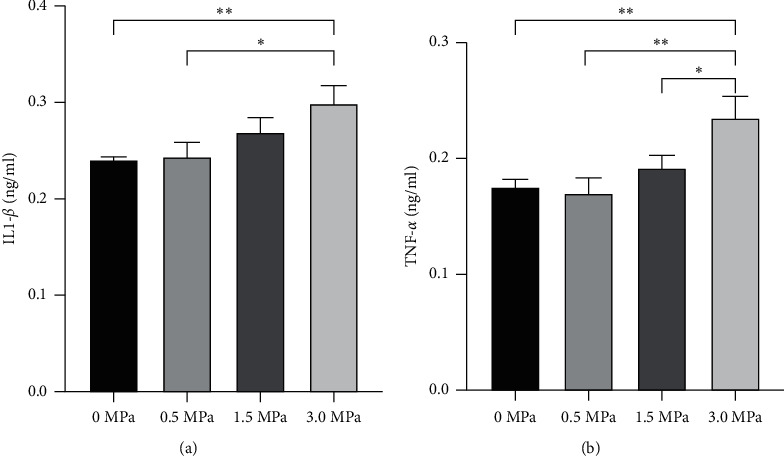
Enzyme-linked immunosorbent assay (ELISA) for detection of the inflammatory factors IL-1*β* (a) and TNF-*α* (b). ^*∗*^*P* < 0.05 and ^∗∗^*P* < 0.01. Each symbol represents the mean ± SEM; one-way ANOVA, followed by LSD test for pairwise comparisons; *n* = 3 rats per assay.

Spinal cord injuries can lead to high expression of inflammatory factors, such as IL-1*β* and TNF-*α*. According to the ELISA results, stimulation with 0.5 MPa and 1.5 MPa LIFU did not significantly increase the expression of IL-1*β* and TNF-*α* compared to the negative stimulation (0 MPa). In contrast, 3.0 MPa stimulation significantly increased the expression of IL-1*β* and TNF-*α* compared to 0 MPa and 0.5 MPa irradiation intensities (*P* < 0.05) (Figure 6).

**Figure 7:**
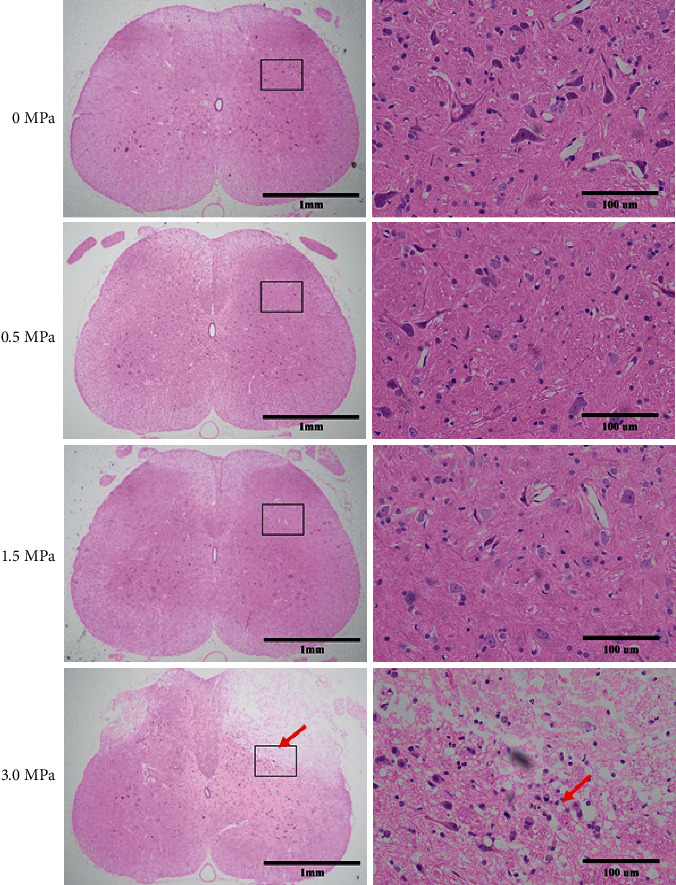
Hematoxylin and eosin (H&E) staining for histological examination of spinal cord injury (×40 and ×400). Scale bars = 1 mm and 100 *μ*m. Comparison of H&E staining among the spinal cord sections after different irradiation intensities of LIFU stimulation. There was no significant difference in histological results among 0 MPa, 0.5 MPa, and 1.5 MPa stimulation groups. In the 3.0 MPa stimulation group, coagulative necrosis was clear at the dorsal horn of the spinal cord (red arrow).

**Figure 8:**
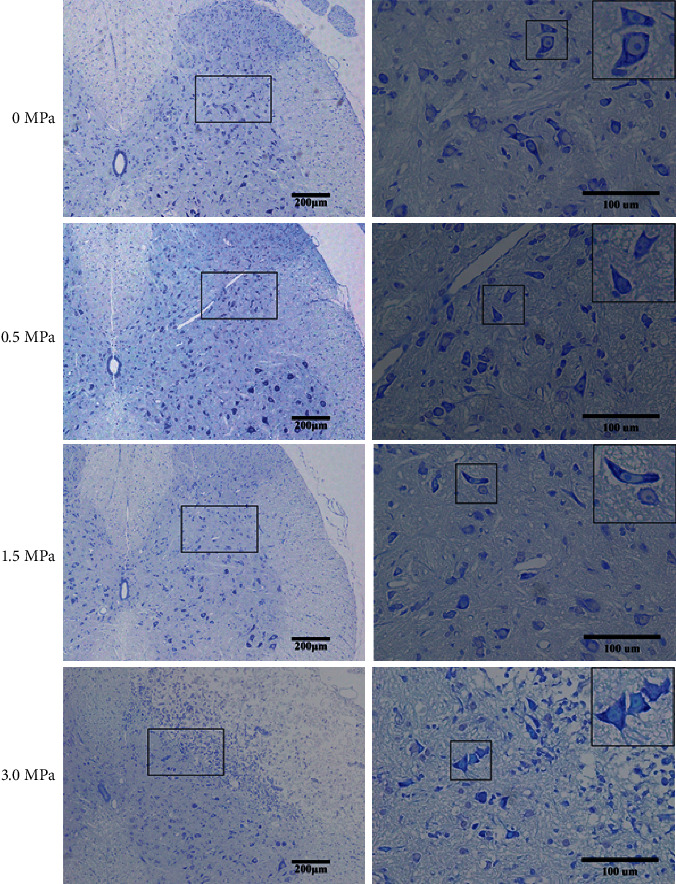
Nissl staining for histological examination of spinal cord injury (×100 and ×400). Scale bars = 200 *μ*m and 100 *μ*m. Comparison of Nissl staining among the spinal cord sections after different irradiation intensities of LIFU stimulation. After 0 MPa, 0.5 MPa, and 1.5 MPa stimulation, the neuron arrangement was regular, the structure was clear, the morphology was normal, and the Nissl body was clear (shown in the black square). After 3.0 MPa irradiation intensity stimulation, the neuron arrangement was irregular, the structure was unclear, the morphology was abnormal, and the Nissl body was unclear (shown in the black square).

**Figure 9:**
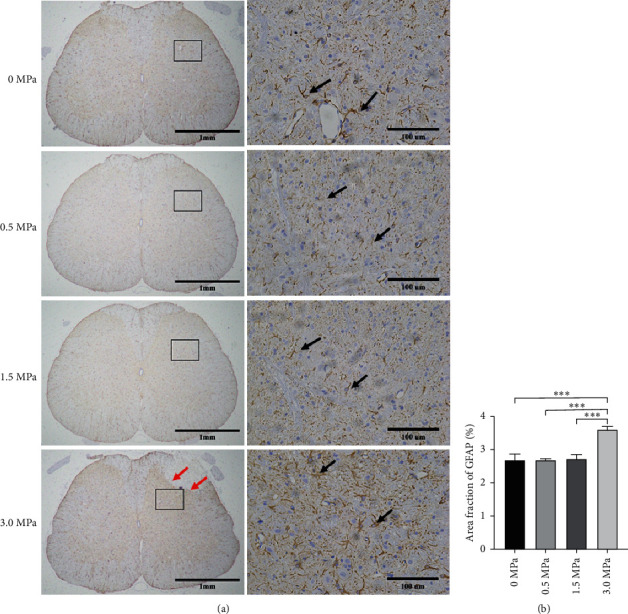
Results of immunohistochemistry (IHC) staining of astrocyte activation in the spinal cord sections after different irradiation intensities of LIFU stimulation (×40 and ×400). Scale bars = 1 mm and 100 *μ*m. (a) Black arrows show the GFAP-positive astrocytes, and the red arrow shows coagulative necrosis of the spinal cord. A diagram indicating a 300 *µ*m × 300 *µ*m square area was defined for further analysis of the positive cells. (b) Intensity analysis of the GFAP-positive area using ImageJ showed that 3.0 MPa stimulation increased the intensity of GFAP. ^∗∗∗∗^*P* < 0.0001. Each symbol represents the mean ± SEM; one-way ANOVA, followed by LSD test for pairwise comparisons; *n* = 3 rats per assay.

Among the LIFU^0+^, LIFU^1+^, and LIFU^2+^ groups, we found no erythrocyte exudation (or bleeding), immune cell infiltration, or coagulative necrosis in H and E staining, no decrease in the numbers of Nissl bodies in Nissl staining, and no significant difference in the fluorescence intensity of GFAP (Figures 7–9). However, in the LIFU^3+^ group, H&E staining revealed coagulation necrosis of the dorsal horn, especially at the right lateral portion (Figure 7). In the LIFU^3+^ group, Nissl staining also showed significant necrosis on the right side of the dorsal horn, with structural destruction and loss of neurons, and the Nissl bodies showed condensation and darker staining (Figure 8). In the LIFU^3+^ group, the intensity of GFAP also increased compared to that of the LIFU^0+^, LIFU^1+^, and LIFU^2+^ groups (*P* < 0.05) (Figure 9).

### 3.5. Western Blot

**Figure 10:**
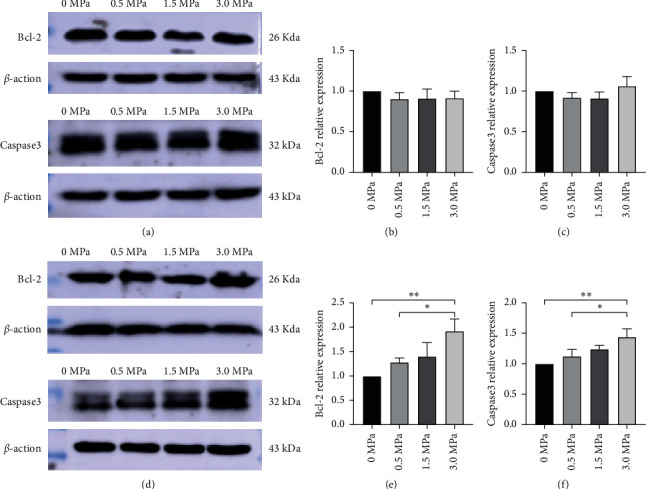
LIFU stimulation induces the expression of caspase-3 and Bcl-2 as markers of apoptosis induction and apoptosis inhibition. (a–c) Western blot results showed that LIFU stimulation did not significantly alter the expression levels of caspase-3 and Bcl-2 on day 0 (*P* > 0.05), while (d–f) 3.0 MPa irradiation intensity stimulation increased the expression levels of caspase-3 and Bcl-2 compared to 0 MPa and 0.5 MPa irradiation intensities on day 3 after LIFU stimulation (*P* < 0.05). There were no significant differences among the 0 MPa, 0.5 MPa, and 1.5 MPa irradiation intensity stimulation groups (*P* > 0.05). Values were normalized to *β*-actin. Each symbol represents the mean ± SEM; ^*∗*^*P* < 0.05, ^∗∗^*P* < 0.01; one-way ANOVA, followed by LSD test for pairwise comparisons; *n* = 3 rats per assay.

We also examined the expression levels of caspase-3 and Bcl-2 as proapoptotic and antiapoptotic markers, respectively. According to the results of western blotting, the expression levels of caspase-3 and Bcl-2 remained unchanged on day 0 after different irradiation intensity stimulations (*P* > 0.05). On day 3 after LIFU stimulation, the 3.0 MPa stimulation significantly increased the expression of caspase-3 and Bcl-2 compared to 0 MPa and 0.5 MPa stimulations (*P* < 0.05), while there was no significant difference among 0 MPa, 0.5 MPa, and 1.5 MPa stimulations (*P* > 0.05) (Figure 10).

## 4. Discussion

After Wall and Melzack [38] first proposed the concept that “control of pain may be achieved by selectively activating the large, rapidly conducting fibers,” spinal cord stimulation has evolved significantly over the past decades [39]. Clinically, spinal cord stimulation or neuromodulation has attracted much attention in the management of chronic spinal conditions, especially for chronic spinal-related pain, such as failed back surgery syndrome/postlaminectomy syndrome, complex regional pain syndrome, and peripheral neuropathic pain [28]. In the past, spinal cord stimulation was induced by electrodes placed in the epidural space with pulse currents of different stimulation modalities, including high-frequency technology, bust stimulation, or other paradigms [40–43]. The electric field formed by electrical stimulation between the electrodes can transfer a specific amount of charge, thus altering the neuronal membrane potential, which is the basis of nerve recruitment [44]. The current study is the first to explore spinal cord neuromodulation with percutaneous LIFU stimulation.

The previous study found that LIFU stimulation of the brain or peripheral nerves can elicit electrophysiological changes [20]. For example, LIFU stimulation of the L5 dorsal root ganglion (DRG) or applied to a peripheral nerve in situ can also alter nerve function, including an increase in mechanical and thermal thresholds and suppression of compound action potentials and sensory action potentials in a neuropathic pain model [45, 46]. Moreover, transcranial LIFU stimulation of the motor cortex activates neurons and evokes motor behavior, and the muscle contraction of limbs has been verified by EMG [20, 21]. In this study, we successfully activated the neurocircuits of the spinal cord by percutaneous stimulation with LIFU when the intensity was >0.5 MPa. Spinal cord activation also induced the recruitment of Sol muscle as measured by EMG.

A previous study has shown that low-intensity ultrasound stimulation can also induce changes in biomarkers. For example, low-intensity ultrasound stimulation of peripheral nerves promoted injured nerve regeneration by stimulating the release of brain-derived neurotrophic factor (BDNF) [47]. Furthermore, transcranial focused ultrasound can decrease the expression of c-fos and increase the expression of GAD65 in the brains of SD rats with epilepsy, indicating that focused ultrasound deactivates excitatory cells and activates GABAergic terminals [37]. Another study has demonstrated that spinal cord activation increases the expression of c-fos and produces analgesia [36]. The increased numbers of c-fos- and GAD65-positive cells indicated the activation of neurons and synapses [36, 37]. In this study, we found that transdermal LIFU increased the numbers of c-fos- and GAD65-positive cells. From the results of EMG and biomarker studies (c-fos and GAD65), we hypothesize that the spinal cord was activated by LIFU.

Our results showed some differences from those of Chen, which showed that transcranial focused ultrasound reduced the number of c-fos-positive cells [37]; the difference may reflect the different animal models adopted. Tissue injury, such as paw inflammation [48], sciatic nerve transection [49], and chronic constriction injury of the sciatic nerve [50], can also increase c-fos expression. A previous study found that the expression of c-fos increased in rats with seizures [51]. However, in this study, normal rats were used, and spinal cord activation by LIFU increased the number of c-fos-positive cells.

Previous studies have shown the many advantages of focused ultrasound stimulation, including high spatial resolution, noninvasive neurostimulation, and effective stimulation of the deep tissues with submillimeter static resolution [7, 8]. Moreover, it has been demonstrated that focused ultrasound can activate cells, brain histology slices, and neurocircuits of nonprimates, primates, and even humans [13, 16, 19, 20, 22]. However, the spinal cord is surrounded by irregular vertebral bones, which could lead to reflection, refraction, diffusion, and absorption of ultrasound beams, all of which may affect the neuromodulatory effects of ultrasound. In this study, we first demonstrated that LIFU enhances Sol muscle contractions and increases the number of c-fos- and GAD65-positive cells, suggesting activation of the spinal cord neurocircuits. The activation of the spinal cord neurocircuits by ultrasound may be due to the use of focused ultrasound, which can converge ultrasound beams onto the target to produce tissue effects [52]. Clearly, the activation of the spinal cord neurocircuits by low-intensity focused ultrasound would also provide an innovative and noninvasive neuromodulation method for spinal cord stimulation.

Safety is an important consideration in neuromodulation. Previous studies have demonstrated that ultrasound can induce different biological effects depending on the exposure parameters; for example, low-intensity ultrasound produces reversible cellular effects, whereas high-intensity ultrasound leads to irreversible cell death. According to the Food and Drug Administration (FDA), the *I*_spta_ of the diagnostic ultrasound must be ≤720 mw/cm^2^ [53], which has been recognized as the requirement for ultrasonic neuromodulation treatment protocols. The safety of the FDA-recommended limit of ultrasound intensity has been demonstrated by previous studies. Some studies have shown that ultrasound intensity above the FDA limits but below the International Electrotechnical Commission (IEC) set intensity (*I*_spta_ = 3 W/cm^2^) for diagnostic medical ultrasound equipment is still safe [54]. Focused ultrasound with an intensity of 3.2 MPa has also been used to stimulate the peripheral nervous system of the mice, activation of which was detected by EMG, while histological examination showed no evidence of nerve damage [55]. In this study, the neuromotor function, electrophysiology, H&E staining, Nissl staining, and protein expression analyses suggested no injury to the spinal cord after 0.5 MPa or 1.5 MPa stimulation. Moreover, ultrasound has little thermal effect and does not cause tissue injury [10, 11]. However, in this study, 3 MPa stimulation decreased the SEP amplitude and increased the expression levels of IL-1*β*, TNF-*α*, caspase-3 and Bcl-2, and GFAP, but it did not affect the neuromotor function or latency of SEPs and MEPs. The decreased amplitude of SEPs indicates damage to the spinal cord sensory pathways. The electrophysiological results were consistent with the H&E staining results, which showed coagulative necrosis of the dorsal horn. Furthermore, the WB results showed increased expression of caspase-3, confirming the apoptosis of neurons after spinal cord injury. The increased expression of Bcl-2 suggests that the spinal cord produced an antioxidant response following injury. The differences observed between this study and previous studies may be due to various factors. First, focused ultrasound forms a focal spot in the deep tissue, which has a higher acoustic pressure and stimulation intensity and can produce stronger biological effects. Second, the spinal cord may exhibit a lower tolerance to ultrasonic stimulation than the peripheral nervous system. Finally, we suggest that the irradiation intensity should be strictly controlled when using low-intensity focused ultrasound to stimulate the central nervous system to avoid irreversible neural damage.

## 5. Limitations

This study has several limitations that warrant discussion. First, although we found that percutaneous low-intensity focused ultrasound activates spinal cord neurocircuits, the detailed mechanism is still unknown. Second, the prestudy confirmed that the temperature change was less than 0.025°C after LIFU stimulation. However, focused ultrasound can lead to spinal cord injury at an irradiation intensity of 3 MPa, and further experiments are needed to clarify the mechanisms underlying spinal cord injuries. Third, we confirmed that LIFU stimulation activates spinal cord neurocircuits, but we did not extend this research to determine how long the effects of LIFU stimulation are maintained.

## 6. Conclusions

Noninvasive LIFU can effectively activate spinal cord neurocircuits and represents a safe neuromodulation method for spinal cord stimulation when the radiation intensity is <1.5 MPa.

## Data Availability

The data used to support the findings of this study are included within the article.

## Abbreviations

LIFU: Low-intensity focused ultrasound
EMG: Electromyography
RI: Radiation intensity
BBB scale: Basso–Beattie–Bresnahan locomotor rating scale
Sol: Soleus
SEPs: Somatosensory evoked potentials
MEPs: Motor evoked potentials
Amp: Amplitude
H&E: Hematoxylin and eosin.

## Supplementary Materials

Supplementary Materials Supplementary Figure 1: the amplitude of EMG after different irradiation intensity stimulations. ^*∗*^*P* < 0.05 and ^∗∗∗∗^*P* < 0.0001. Each symbol represents the mean ± SEM; one-way ANOVA, followed by LSD test for pairwise comparisons; *n* = 6 rats per assay. Supplementary Figure 2: (a) the Basso, Beattie, and Bresnahan locomotor rating scale (BBB scale) was used to assess the neuromotor function of the hind limbs and the latency of (b) somatosensory evoked potentials (SEPs) and (d) motor evoked potentials (MEPs) evaluation for the spinal cord. The results show no difference in neuromotor function, latency of SEPs, and latency of MEPs after the different irradiation intensity stimulation (*P* > 0.05). (c) MEPs were used to detect motor conduction for the spinal cord, and the triangle represents the excitation point time. Each symbol represents the mean ± SEM; paired *t*-test; *n* = 6 rat per assay.
