# Supplementary material for: Effects of Noninvasive Low-Intensity Focus Ultrasound Neuromodulation on Spinal Cord Neurocircuits In Vivo
Source: Evid Based Complement Alternat Med. 2021 Nov 27;2021:8534466. doi: 10.1155/2021/8534466 (PMC8643243; doi:10.1155/2021/8534466)
Supplement: Supplementary Materials — Supplementary Figure 1: the amplitude of EMG after different irradiation intensity stimulations. ∗P < 0.05 and ∗∗∗∗P < 0.0001. Each symbol represents the mean ± SEM; one-way ANOVA, followed by LSD test for pairwise comparisons; n = 6 rats per assay. Supplementary Figure 2: (a) the Basso, Beattie, and Bresnahan locomotor rating scale (BBB scale) was used to assess the neuromotor function of the hind limbs and the latency of (b) somatosensory evoked potentials (SEPs) and (d) motor evoked potentials (MEPs) evaluation for the spinal cord. The results show no difference in neuromotor function, latency of SEPs, and latency of MEPs after the different irradiation intensity stimulation (P > 0.05). (c) MEPs were used to detect motor conduction for the spinal cord, and the triangle represents the excitation point time. Each symbol represents the mean ± SEM; paired t-test; n = 6 rat per assay. [file 8534466.f1.docx]

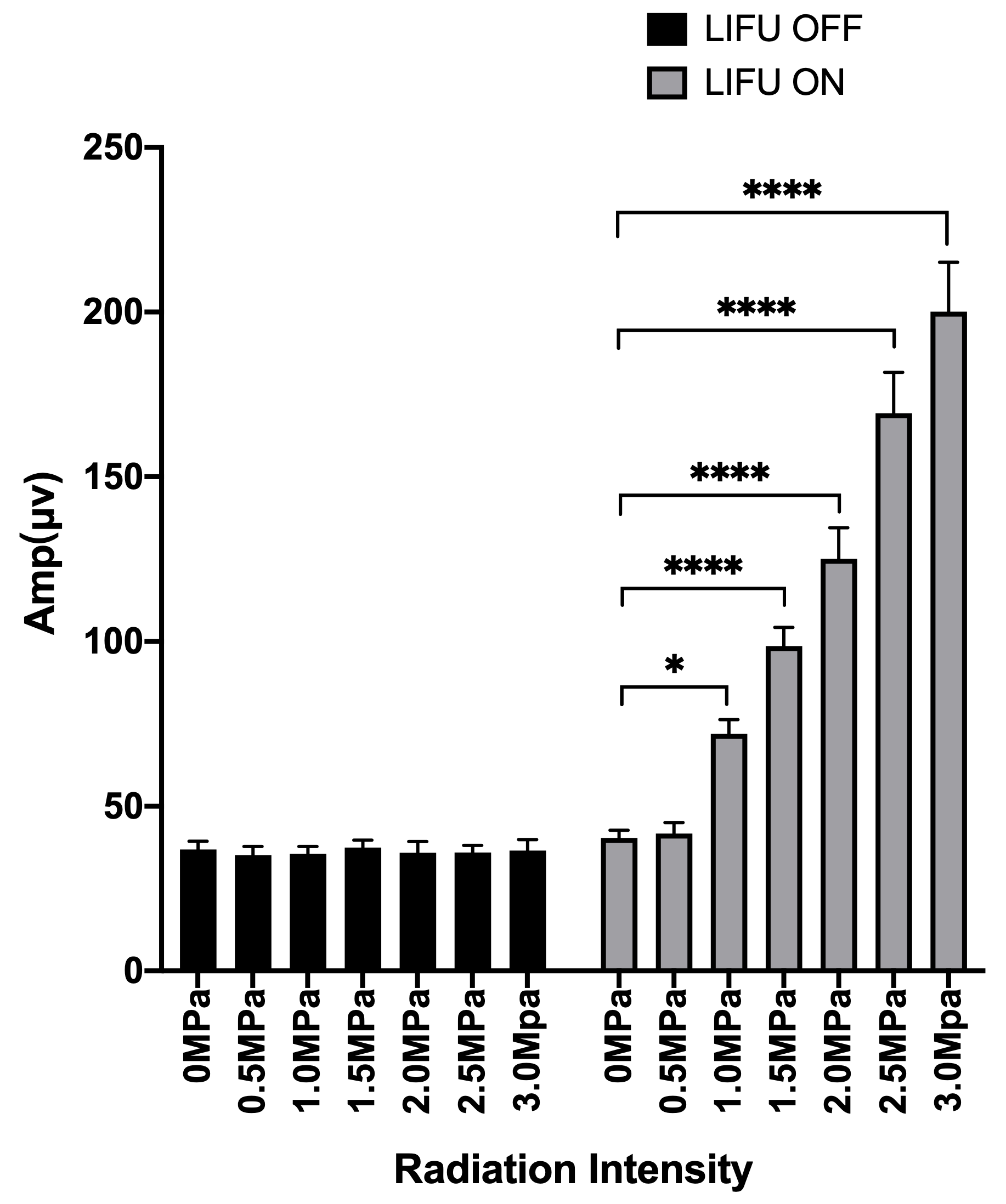


**Supplementary Figure 1**: The amplitude of EMG after different irradiation intensity stimulations. *P < 0.05, ****P < 0.0001. Each symbol represents the mean ± SEM; One-way ANOVA, followed by LSD test for pairwise comparisons; n = 6 rats per assay.

(d)


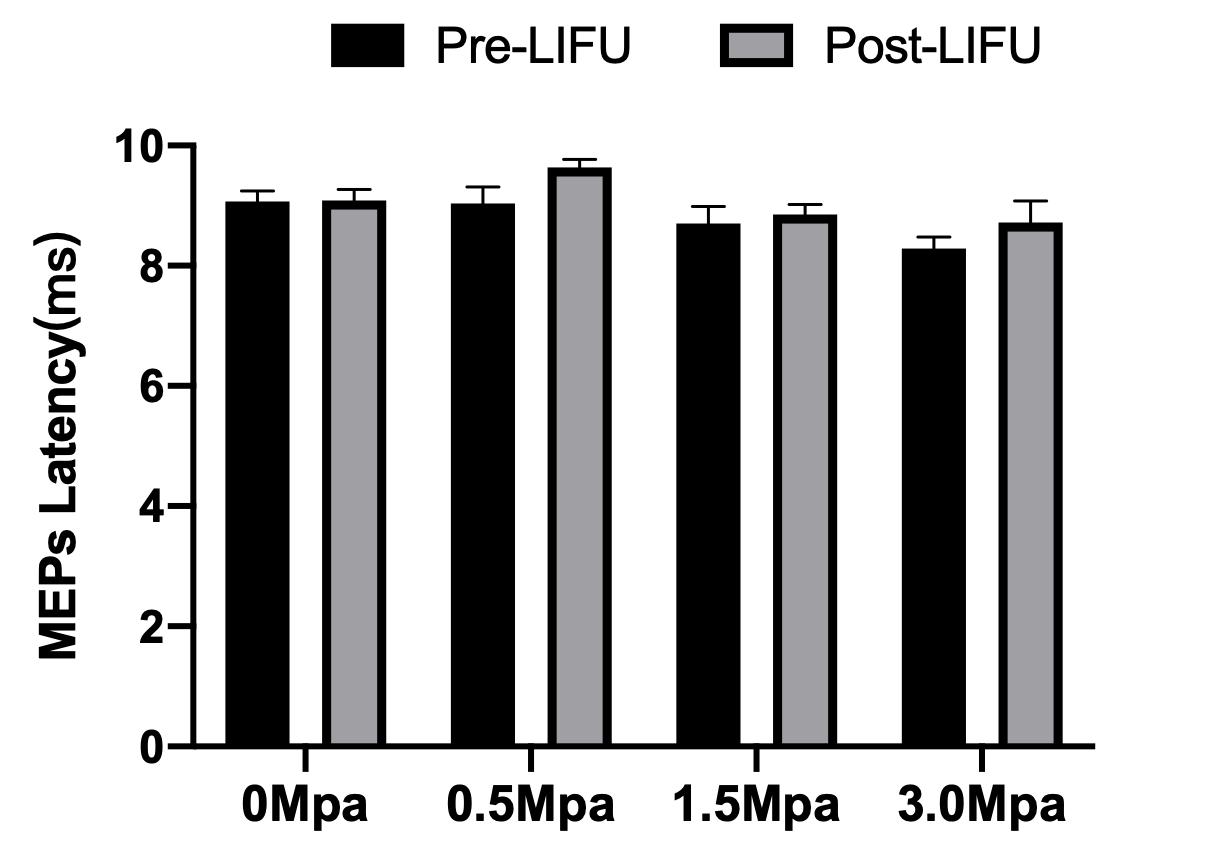


(c)


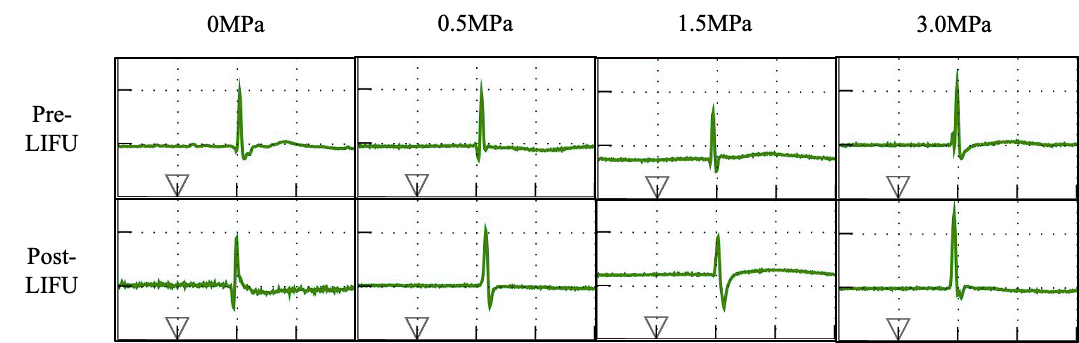


(b)


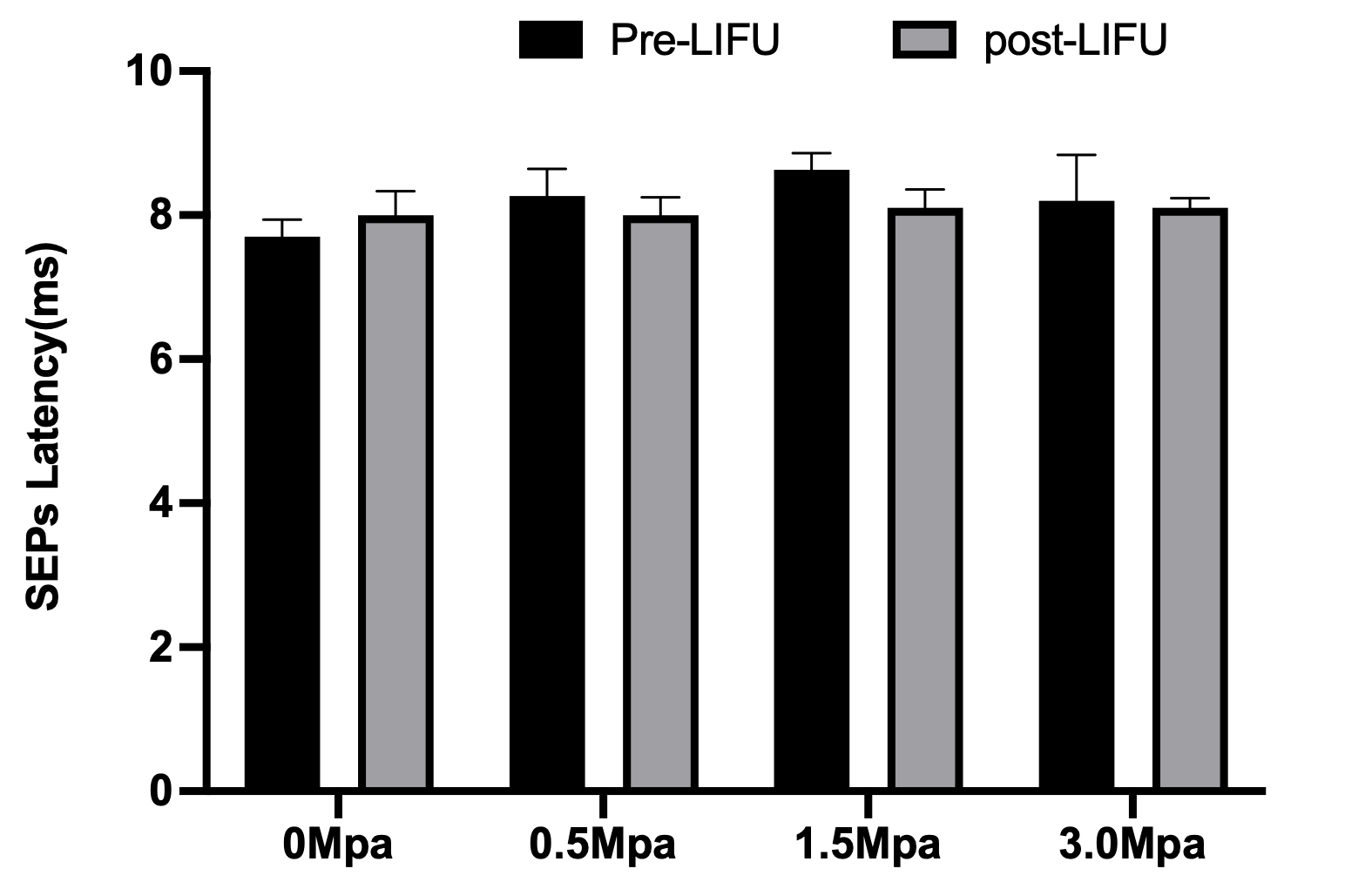


(a)


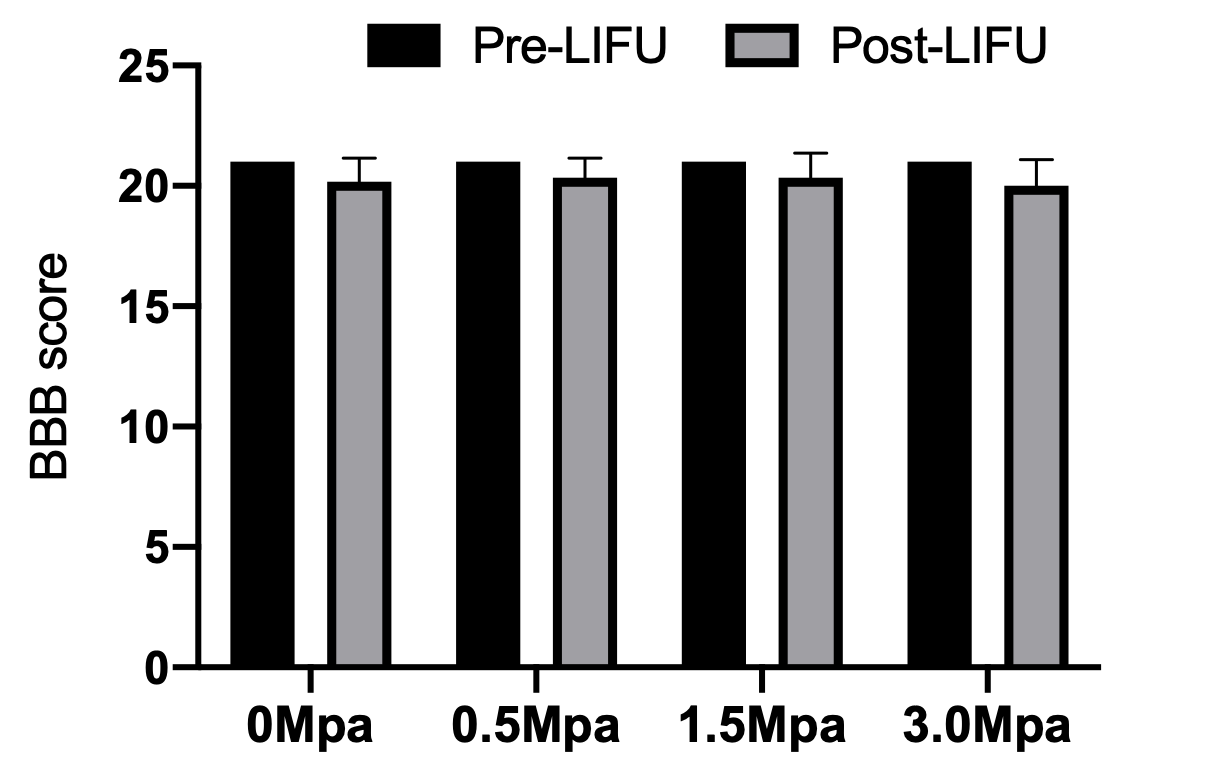


**Supplementary Figure 2**: **(a)** The Basso, Beattie & Bresnahan locomotor rating scale (BBB scale) was used to assess the Neuromotor function of hind limbs, and Latency of **(b)** Somatosensory Evoked Potentials (SEPs) and **(d)** motor evoked potentials **(**MEPs) evaluation for Spinal cord, the results shown no difference about Neuromotor function, Latency of SEPs, and Latency of MEPs after the different irradiation intensity stimulation (P>0.05). **(c)** MEPs was used to detect motor conduction for the spinal cord, and the triangle is present the excitation point time. Each symbol represents the mean ± SEM; Paired *t* test; n=6 rat per assay.
